# Does Abdominal Obesity Accelerate Muscle Strength Decline in Older Adults? Evidence From the English Longitudinal Study of Ageing

**DOI:** 10.1093/gerona/gly178

**Published:** 2018-08-10

**Authors:** Danilo Henrique Trevisan de Carvalho, Shaun Scholes, Jair Licio Ferreira Santos, Cesar de Oliveira, Tiago da Silva Alexandre

**Affiliations:** 1 Department of Physical Therapy, Federal University of Sao Carlos, Brazil; 2 Department of Epidemiology and Public Health, University College London, UK; 3 Department of Social Medicine, University of Sao Paulo, Ribeirao Preto, Brazil; 4 Department of Gerontology, Federal University of Sao Carlos, Brazil

**Keywords:** Grip strength, Waist circumference, Longitudinal, Trajectories

## Abstract

**Background:**

Cross-sectional evidence has shown an association between abdominal obesity and lower muscle strength in older adults. However, no longitudinal findings have confirmed this association. In addition, the impact of abdominal fat on the reduction in muscle strength is not yet fully understood.

**Methods:**

We investigated the longitudinal associations between abdominal obesity and handgrip strength in 5,181 older adults from the English Longitudinal Study of Ageing over 8 years of follow-up. Muscular strength was measured using a manual dynamometer. Abdominal obesity was defined as a waist circumference >102 cm for men and >88 cm for women. Generalized linear mixed models were adjusted by measures of socioeconomic status, health conditions, lifestyle, cognition, depressive symptoms, biomarkers, and disability.

**Results:**

At baseline, the mean age of participants was 65.8 years and their mean waist circumference and body mass index (BMI) were 95 cm and 27.7 kg/m^2^, respectively. Fully adjusted models showed that abdominal obese men and women had stronger muscle strength at baseline. The decline over time in muscle strength was accelerated in abdominal obese men (−0.12 kg/year, 95% confidence interval: −0.24 to −0.01) compared with nonabdominal obese. This association was not found in women. Comparative analyses showed that overweight men according to their BMI were not at greater risk of muscle strength decline. However, these men were at risk based on their waist circumference.

**Conclusions:**

Abdominal obesity is associated with accelerated muscle strength decline in men.

Muscle strength and muscle mass decline with aging. The decline is faster for muscle strength, and it is strongly associated with disability and death (1,2). Gender differences are found in terms of absolute levels of muscle strength as well as loss of strength over time, both greater among men (1,3,4). However, the mechanisms underlying these differences and the effect on functional muscle properties are not yet fully understood (1,4).

Musculoskeletal aging has been accompanied by increasing obesity in older adults globally (5). The relationship between muscle strength and adiposity has been explored, but the strength of this association varies according to the anthropometric measure adopted, that is, body mass index (BMI) or waist circumference. Cross-sectional findings from 8,441 participants from the European Prospective Investigation into Cancer-Norfolk Study (4) showed that higher BMI was associated with stronger grip strength, but a larger waist circumference was associated with a weaker handgrip.

Recent evidence shows that abdominal fat contributes to a greater loss of muscle strength via neuroendocrine dysregulations (3,6). In addition, there are gender differences related to abdominal fat deposition patterns. Women accumulate more subcutaneous fat, whereas men accumulate more visceral fat (5). Such differences could also play a role in the different speed of muscle strength decline between men and women.

To the best of our knowledge, this is the first longitudinal study to investigate whether abdominal obesity accelerates muscle strength decline among older adults over a period of 8 years of follow-up.

## Methods

### Study Population

The English Longitudinal Study of Ageing (ELSA) is an ongoing longitudinal panel study of community-dwelling people aged 50 years and older in England that commenced in 2002. ELSA sample was drawn from participants who had previously participated in the Health Survey for England (HSE) (7), an annual health examination survey that each year recruits a different nationally representative sample using a multistage stratified random probability design. After baseline, follow-up interviews within ELSA occur every 2 years and health examinations, that is, a nurse visit, every 4 years. The first health examination was in 2004–2005. A detailed description of the study can be found elsewhere (7). Analyses for this study included longitudinal data from Waves 2 (2004–2005), 4 (2008–2009), and 6 (2012–2013). All ELSA participants gave written informed consent. The National Research and Ethics Committee granted Ethical approval for all the ELSA waves (MREC/01/2/91).

### Assessment of Muscle Strength

Grip strength (kilograms) was measured three times for each hand using the Smedley dynamometer. During the test, the participant remained standing with the arm alongside the trunk and elbow at 90 degrees (8). Three maximum strength tests were performed with a 1-minute rest between tests, and the highest strength value in the dominant hand was used in our analysis.

### Waist Circumference Assessment

Waist circumference (centimeters) was determined using a flexible metric tape at the midpoint between the iliac crest and last rib. The participant remained standing with arms alongside the body and the trunk free of clothing. The measurement was performed with the abdomen relaxed at the end of expiration (8). Abdominal obesity was defined as a waist circumference >88 cm for women and >102 cm for men (9).

### Covariates

Sociodemographic covariates were age (years), marital status (with or without conjugal life), household wealth (quintiles), and education level (categorized using number of years of schooling as follows: 0–11, 12–13, >13).

Behavioral characteristics included cigarette smoking (nonsmoker, former smoker, or current smoker), alcohol intake (weekly frequency: ≤1 day, 2–6 days, daily), and physical activity level (classified based on the practice of vigorous, moderate, low-intensity physical activities, or none at least once a week) (10).

Health conditions included self-reported arthritis, cancer, heart disease, stroke, lung disease, and falls (within the last 2 years). Individuals with self-reported hypertension and/or mean systolic pressure ≥140 mmHg and/or diastolic ≥90 mmHg were considered hypertensive (11). Individuals with self-reported diabetes and/or those with plasma glucose ≥126 mg/dL and/or glycated hemoglobin ≥6.5% were considered diabetic (12). Women with hemoglobin <12 mg/dL and men <13 mg/dL were considered anemic (13). Depressive symptoms were defined by the Center for Epidemiologic Studies Depression Scale (CES-D) score ≥4 (14). Memory score, our marker of cognitive function, was based on the summation of immediate and delayed-recall results from a 10 word-list learning test (score range: 0–20) (15).

BMI was calculated by dividing mass in kilograms by height in square meters (kg/m^2^) and classified as follows: normal weight (18.5–24.9 kg/m^2^), underweight (<18.5 kg/m^2^), overweight (25.0–29.9 kg/m^2^), and obese (≥30 kg/m^2^) (16). Because the relationship between muscle strength decline and abdominal obesity could be affected by weight change, particularly weight loss, we created a variable based on weight change between waves to adjust our models. This variable was categorized as follows: stable weight compared with previous wave, weight loss equal or superior to 5% compared with previous wave, and weight gain equal or superior to 5% compared with previous wave.

Blood measures: participants with hematological disorders, those who took anticoagulants, and those who declined to provide consent did not participate in the blood collection. Further information on the laboratory analyses are found elsewhere (17). The cutoff points for the biomarkers were as follows: triglycerides (≥150 mg/dL), total cholesterol (≥200 mg/dL), HDL (<40 mg/dL for men and <50 mg/dL for women), LDL (≥100 mg/dL) (18), C-reactive protein (≥3 mg/L), fibrinogen (>3.8 g/L) (19), and ferritin (<39 ng/mL for women and <62 ng/mL for men) (8). Disability: Physical functioning was measured using self-reported limitations in the following basic activities of daily living: dressing, walking across a room, bathing or showering, eating, getting in or out of bed, using the toilet; and in the following instrumental activities of daily living: making telephone calls, shopping for groceries, preparing hot meals, doing work around the house or garden, taking medications, and managing money, such as paying bills and keeping track of expenses. Both variables were used quantitatively based on the number of limitations (range: activities of daily living 0–6; instrumental activities of daily living 0–7) (20).

### Statistical Analysis

Baseline characteristics were expressed as means and proportions. Differences between (a) included and excluded individuals (due to missing data) and (b) gender and abdominal obesity status were analyzed using chi-square tests, analysis of variance, and post hoc Tukey tests. A *p* value <.05 was used to indicate statistical significance.

Generalized linear mixed models were performed to estimate the trajectories in muscle strength decline in nonabdominal obese and abdominal obese participants, using the XTMIXED command of STATA 14 (StataCorp, College Station, TX). These models best handle unbalanced data from studies with repeated measures and enable the statistical modeling of changes in a time-dependent outcome variable (handgrip strength), as well as allowing time-dependent change in the magnitude of association between variables (21,22).

Because of significant differences in the intercept and slope values for muscle strength between men and women (*p* < .01) and also gender differences in the rate of muscle strength decline (1,3), all analyses were stratified by gender according to abdominal obesity status. The two final models, that is, one for each gender, included the interaction between time (denoting years of follow-up) and abdominal obesity status adjusted by all the covariates previously described.

The intercept represents the estimated mean difference in muscle strength between individuals with and without abdominal obesity (reference category) at baseline. On the slope, time (in years) indicates whether the trajectory in muscle strength decline occurs independently of the presence of covariates (whether time per se is a determinant for this decline). The time by abdominal obesity interaction (Time × Abdominal Obese) represents the estimated difference in the annual rate of change (slope) between abdominal obese participants and the reference (nonabdominal obese) in muscle strength decline. The decline rates in grip strength were compared using β coefficients and 95% confidence intervals.

Two comparative analyses stratified by gender were performed. First, we tested whether muscle strength decline over time for overweight or obese participants according to BMI differed from the one observed according to abdominal obesity, that is, waist circumference. The other comparative analysis, tested as the main outcome grip strength normalized by BMI values as a function of abdominal obesity status.

## Results

Of the 7,666 individuals who participated in the baseline health examination (2004–2005), 2,485 were excluded due to missing data on grip strength, waist circumference, or covariates, resulting in a final sample of 5,181 individuals. Of this final sample, 4,026 and 3,511 were reassessed at 4 and 8 years of follow-up, respectively.

Table 1 shows the characteristics of participants at baseline according to sex and abdominal obesity status. Participants excluded due to missing data were older, weaker, more abdominal obese, and more functionally compromised than those included in present study. They also had lower levels of education and wealth, had less conjugal life and hypertension, smoked more, and reported more sedentary lifestyle, anaemia, diabetes, arthritis, cancer, stroke, heart disease, falls, and depressive symptoms than those included. In addition, excluded participants had higher concentrations of C-reactive protein (CRP) and triglycerides as well as lower concentrations of high-density lipoprotein (HDL) and low-density lipoprotein (LDL) (*p* < .05; Supplementary Table 1).

**Table 1.** Baseline Characteristics by Gender and Abdominal Obesity Status of 5,181 Older Adults From the English Longitudinal Study of Aging (2004–2005)

	Women		Men	
	(*n* = 2,827) 54.6%		(*n* = 2,354) 45.4%	
	Non-abdominal Obese (*n* = 1,302)	Abdominal Obese (*n* = 1,525)	Non-abdominal Obese (*n* = 1,345)	Abdominal Obese (*n* = 1,009)
Age, years	65.8 (9.7)	66.0 (9.3)	65.7 (9.2)	65.7 (9.0)
Marital status (with conjugal life), (%)	61.2*	61.9*	75.5^†^*	79.9^†^*
Wealth (quintiles), (%)				
Lowest quintile	27.1^†^	18.7^†^*	26.4^†^	21.3^†^*
Second quintile	21.7^†^	19.2^†^*	25.2^†^	23.0^†^*
Third quintile	19.3^†^	21.7^†^*	19.6^†^	22.0^†^*
Fourth quintile	17.4^†^	20.4^†^*	16.2^†^	19.1^†^*
Highest quintile	13.5^†^	18.0^†^*	11.5^†^	13.7^†^*
Not declared	1.0^†^	2.0^†^*	1.1^†^	0.9^†^*
Schooling, (%)				
0–11 years	50.0^†^*	57.2^†^*	39.8^†^*	46.8^†^*
12–13 years	26.9^†^*	24.3^†^*	24.8^†^*	24.8^†^*
>13 years	23.1^†^*	18.5^†^*	35.4^†^*	28.4^†^*
Physical activity level, (%)				
Sedentary	3.1^†^*	3.8^†^*	2.9^†^*	3.6^†^*
Low	12.8^†^*	20.5^†^*	8.3^†^*	15.0^†^*
Moderate	51.8^†^*	53.0^†^*	49.5^†^*	52.2^†^*
Vigorous	32.3^†^*	22.7^†^*	39.3^†^*	29.2^†^*
Alcohol intake, (%)				
≤1 day	38.1^†^*	45.0^†^*	21.0^†^*	26.9^†^*
2–6 days	39.6^†^*	35.0^†^*	48.0^†^*	44.5^†^*
Daily	14.8^†^*	11.9^†^*	22.7^†^*	18.6^†^*
Not declared	7.5^†^*	8.1^†^*	8.3^†^*	10.0^†^*
Smoking, (%)				
Non-smoker	47.0^†^*	43.1^†^*	31.0^†^*	25.3^†^*
Former smoker	39.5^†^*	43.6^†^*	53.5^†^*	63.1^†^*
Current smoker	13.5^†^*	13.3^†^*	15.5^†^*	11.6^†^*
Stroke (yes), (%)	1.1	1.2	1.1	0.9
Anaemia (yes), (%)	6.4^†^	4.3^†^	5.6	5.0
Cancer (yes), (%)	2.7	2.4*	2.0^†^	4.2^†^*
Heart disease (yes), (%)	6.8	8.2	7.3	7.9
Diabetes (yes), (%)	1.0^†^*	4.8^†^*	4.1^†^*	7.4^†^*
Arthritis (yes), (%)	33.9^†^*	45.2^†^*	22.7^†^*	31.5^†^*
Hypertension (yes), (%)	36.5^†^	48.1^†^	39.4	49.4
Osteoporosis (yes), (%)	10.9^†^*	8.2^†^*	1.3*	2.1*
Lung disease (yes), (%)	11.7^†^	15.5^†^	12.3	15.1
Fall (yes), (%)	22.9^†^*	25.9^†^*	15.1*	16.5*
Cognition (mean), points	10.7 (3.5)^†^*	10.4 (3.4)^†^*	9.9 (3.4)*	9.7 (3.2)*
Depressive symptoms (yes), (%)	14.6^†^*	18.1^†^*	8.5*	10.6*
Handgrip (kg), (mean)	23.8 (6.7)*	24.2 (6.7)*	39.5 (9.7)^†^*	41.1 (10.0)^†^*
Waist circumference (cm), (mean)	79.9 (5.7)^†^*	99.0 (8.9)^†^*	93.4 (6.4)^†^*	110.6 (7.1)^†^*
Body mass index (kg/m^2^), (mean)	24.0 (2.8)^†^*	30.9 (4.6)^†^	25.2 (2.5)^†^*	30.7 (3.2)^†^
Normal weight (%)	61.6^†^*	4.7^†^*	42.5^†^*	0.9^†^*
Underweight (%)	2.2^†^*	0.0^†^*	1.0^†^*	0.0^†^*
Overweight (%)	34.7^†^*	42.7^†^*	54.1^†^*	45.0^†^*
Obese (%)	1.5^†^*	52.6^†^*	2.4^†^*	54.1^†^*
Triglycerides (≥150 mg/dL), (%)	26.6^†^*	48.5^†^*	37.2^†^*	55.8^†^*
Total cholesterol (≥200 mg/dL), (%)	83.0^†^*	78.9^†^*	66.2^†^*	61.4^†^*
HDL (*<*40 mg/dL ♂; <50 mg/dL ♀), (%)	12.5^†^*	26.5^†^*	3.6^†^	9.3^†^*
LDL (≥100 mg/dL), (%)	90.7^†^*	87.5^†^*	82.6^†^	77.2^†^*
C-reactive protein (>3 mg/L), (%)	22.4^†^	49.6^†^*	25.5^†^	41.3^†^*
Fibrinogen (>380 mg/dL), (%)	13.8^†^	21.0^†^*	14.5	16.7*
Ferritin (<39 ng/mL ♂; <62 ng/mL ♀), (%)	22.2^†^	18.7^†^	23.1^†^	17.9^†^
ADL (mean of impairments)	0.2 (0.7)^†^	0.4 (0.9)^†^	0.2 (0.7)^†^	0.4 (0.9)^†^
IADL (mean of impairments)	0.3 (0.8)^†^*	0.4 (0.9)^†^*	0.2 (0.7)*	0.2 (0.7)*

Notes: Data expressed as proportion, mean, and standard deviation. Abbreviations: ADL = basic activities of daily living; HDL = high-density lipoprotein; IADL = instrumental activities of daily living; LDL = low-density lipoprotein.

*Difference between sexes within same abdominal obesity status (*p* < .05).

^†^Difference between states of abdominal obesity within same sex (*p* < .05).

The prevalence of abdominal obesity at baseline was higher among women (53.9%) than men (42.9%). The same occurred at the follow-up: 2008–2009 (60.1% vs 47.4%, respectively) and 2012–2013 (58.7% vs 45.3%, respectively; *p* < .01 for all comparisons).

**Figure 1.**
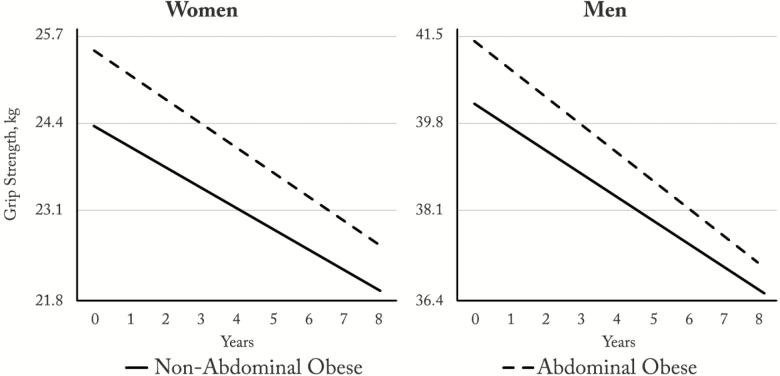
Trajectories of handgrip strength according to abdominal obesity status over time (2004–2005 to 2012–2013). Predictions for individuals aged 50 years; with conjugal life; in highest wealth quintile; with higher level of education; nonsmokers; with little or no alcohol intake; practicing vigorous physical exercise; stable weight; without arthritis, cancer, heart disease, stroke, lung disease, osteoporosis, hypertension, diabetes, anemia, or falls; with CES-D < 4; mean memory score = 20; without functional limitations; and without abnormal concentrations of triglycerides, total cholesterol, LDL, HDL, C-reactive protein, fibrinogen, and ferritin. Abbreviation: CES-D = Center for Epidemiologic Studies Depression Scale.

Table 2 displays the estimated parameters of the generalized linear mixed models for the change in grip strength as a function of abdominal obesity status over the 8-year follow-up period for women and men, respectively. In these models, abdominal obese men and women began at an intercept with greater muscle strength than their nonabdominal obese counterparts (*p* < .01). However, only abdominal obese men had an accelerated decline in strength over time (−0.12 kg/year; 95% confidence interval: −0.24 to −0.01). Moreover, the estimated change over time in muscle strength remained stable in the reference groups in both genders (when all covariates were either zero or mean values). The estimated annual change on handgrip strength values in abdominal and nonabdominal men and women is shown in Figure 1 and Supplementary Table 2.

**Table 2.** Generalized Linear Mixed Models Estimates for Handgrip Values as a Function of Abdominal Obesity Status Over an 8-Year Follow-up Period

	Women	Men
	*n* = 2,827	*n* = 2,354
	Estimated Parameter (95% CI)	
Intercept (baseline)		
Non-abdominal Obese	Reference	Reference
Abdominal Obese	0.84 (0.45–1.23)**	1.56 (0.97–2.15)**
Slope (follow-up)		
Time, years	−0.04 (−0.57–0.50)	−0.58 (−1.29–0.16)
Time × Non-abdominal Obese	Reference	Reference
Time × Abdominal Obese	−0.03 (−0.11–0.06)	−0.12 (−0.24–−0.01)*

Notes: All models adjusted by socioeconomic variables, behavioral characteristics, health conditions, depression, cognition, serum markers, and disability. Abbreviation: CI = confidence interval.

*p < .05.

**p < .01.

The first comparative analysis using only BMI categories (Table 3) revealed similar findings to our main analysis that used only waist circumference as a measure of obesity. We found that obese men (BMI > 30.0 kg/m^2^) start with a greater muscle strength, but their decline over time in muscle strength was faster compared with those with normal weight men. This association was not found among overweight men. An important finding relates to the fact that 45% of abdominal obese men (defined according to waist circumference [WC]) were also overweight (BMI) at baseline (Table 1). Therefore, despite overweight men (BMI) not being identified as at risk of decline in muscle strength, they were at greater risk based on their waist circumference, that is, abdominal obesity.

**Table 3.** Generalized Linear Mixed Models Estimates for Handgrip Values as a Function of Body Mass Index Over an 8-Year Follow-up: A Comparative Analysis

	Women	Men
	*n* = 2,827	*n* = 2,354
	Estimated Parameter (95% CI)	
Intercept (baseline)		
Normal weight	Reference	Reference
Underweight	−0.03 (−1.87–1.80)	−2.92 (−6.58–0.73)
Overweight	1.00 (0.57–1.48)**	1.50 (0.80–2.21)**
Obese	1.47 (0.94–2.01)**	3.26 (2.38–4.14)**
Slope (follow-up)		
Time, years	−0.00 (−0.54–0.53)	−0.44 (−1.17–0.29)
Time × Normal weight	Reference	Reference
Time × Underweight	−0.12 (−0.53–0.28)	0.27 (−0.68–1.23)
Time × Overweight	−0.06 (−0.16–0.03)	−0.03 (−0.16–0.10)
Time × Obese	−0.06 (−0.17–0.04)	−0.17 (−0.33–−0.01)*

Notes: All models adjusted by socioeconomic variables, behavioral characteristics, health conditions, depression, cognition, serum markers, and disability. Abbreviation: CI = confidence interval.

*p < .05.

**p < .01.

The intercept values found in the comparative analysis testing grip strength normalized by BMI values as a function of abdominal obesity status showed that abdominal obese men and women had weaker muscle strength compared with reference category (Supplementary Table 3). These findings are opposite to the ones from the main analysis using handgrip strength absolute values as the main outcome measure. Furthermore, abdominal obese women showed significant annual increases in muscle strength (slope). Therefore, the use of grip strength normalized by BMI is not a good analytical approach as waist circumference and BMI are highly correlated (Pearson’s correlation coefficient = .80; *p* < .05), and, ultimately, both outcome and exposure would have a body fat measure.

## Discussion

In this large population-based cohort of older adults, we demonstrated that older abdominal obese men have a faster rate of decline in muscle strength over 8 years of follow-up, despite their stronger muscle strength at baseline. In addition, abdominal adiposity measured by WC seems to be a better predictor than BMI at identifying individuals at risk of an accelerated loss in muscle strength. In our analyses, the association observed between abdominal obesity and decline in muscle strength was independent of confounders: age, economic circumstances and a wide range of health conditions, lifestyle, biomarkers, physical function, depression, and cognitive function.

Waist circumference is a more adequate measure for the evaluation of adiposity in older adults (23) because body composition in this population can change independently of variations in total mass (3) and BMI can fail to diagnose up to 50% of obesity cases (24).

Our findings differ partially from the ones reported by Koster and colleagues (25), who analyzed longitudinal data from 2,307 men and women (aged 70–79 years) from the Health, Aging, and Body Composition Study. They found that greater total fat mass (evaluated using dual-energy X-ray absorptiometry [DEXA]) was associated with greater muscle strength at baseline in both sexes but did not find that greater fat mass exacerbated the muscle strength decline. However, the quantification of total fat mass in this study (25) does not indicate body fat distribution, which is an important information. The adipose tissue characteristics are not homogeneous, and different consequences may occur depending on the location of fat accumulation. For instance, greater abdominal fat may reflect greater inflammatory activity (5).

In a 22-year follow-up study of 963 men and women (aged 30–73 years), Stenholm and colleagues (26) found that obese individuals (BMI ≥ 30 kg/m^2^) had a more accelerated decline in handgrip strength compared with those in the ideal BMI group. Although obesity classified according to BMI was a predictor of decline in muscle strength, it is not known what this association would be if the analyses were stratified by sex.

Abdominal adiposity consists of subcutaneous and visceral fat with distinct proportions for men and women (5). Men accumulate fat predominantly in the visceral abdominal region, which is strongly associated with elevated expression of proinflammatory cytokines (5,27) and insulin resistance (28). These associations may mediate the accelerated decline in muscle strength observed in abdominal obese men.

The increase in proinflammatory cytokines seems to exacerbate muscle catabolism by raising levels of IL-6, TNF-α, and TNF-β (27); depressing the anabolic process through the increase in TNF-α and TNF-β levels (27,29); and negatively affecting the muscle tissue repair (suppression of IGF-1 repair agent) (30), consequently leading to a greater loss of muscle strength (3,6). Moreover, in the present study, diabetes was more frequent among abdominal obese men at baseline. Insulin resistance may compromise muscle anabolism (28), break down protein for energy purposes, and trigger a greater loss of muscle strength (31).

Visceral fat is also strongly associated with elevated triglycerides levels and fatty infiltration of muscle tissue (32,33). Evidence indicates that intramuscular fat may cause cellular apoptosis resulting in less strength (33). Although no inferences can be made regarding the amount of intramuscular fat in the present study, hypertriglyceridemia was more frequent among abdominal obese men at baseline, and such individuals could also have greater fatty infiltration.

In our sample, the prevalence of abdominal obesity was higher in women, but this fact was not associated with a faster rate of decline in muscle strength. The absence of such an association is not well understood, but characteristics regarding the accumulation of abdominal fat in women seem to help them to have a more favorable muscle strength trajectory in middle to older age compared with men.

Young women have more subcutaneous fat in their hips and thighs. At about the age of 50 years, women undergo an accelerated redistribution of fat to the subcutaneous abdominal region, characterized by less inflammatory activity in comparison with visceral fat and involving greater release of circulating fatty acids (5,32). Although abdominal obese women had higher C-reactive protein and fibrinogen plasma levels at the baseline, these are not good markers of abdominal obesity such as IL-6, TNF-α, and TNF-β (27,29).

A potential explanation for waist circumference not being able to predict a faster rate of muscle strength decline in women could be because women accumulate more subcutaneous fat, whereas men accumulate more visceral fat. Subcutaneous fat might be protective against the dangers of visceral fat.

Future research should investigate the waist-to-hip ratio as a protective measure for this outcome in women because it reflects greater adiposity in the lower trunk and hips (34). This was not done in our study because there were only two measures of this information in the follow-up. The effect of abdominal obesity as a risk factor for worse physical mobility trajectories as well as lower extremity function should also be explored.

Our study has several strengths and potential limitations that need to be acknowledged. The major strength is the use of a large nationally representative sample of community-dwelling English people aged 50 years and older as well as the use of objective health measures (WC, BMI, handgrip strength, and blood analytes). Our analyses included data from three waves and had a long follow-up period. The models were adjusted by a wide range of important covariates associated with both outcome and exposure. Finally, additional analyses were performed to address any potential questions about the obesity measure used. The findings from the comparative analyses support the hypothesis that greater abdominal fat is an important predictor of decline in muscle strength in men.

Limitations arise from the fact that participants excluded from our analyses due to missing data at baseline were older and weaker, and had more abdominal obesity. Therefore, the association between abdominal obesity and accelerated muscle strength decline may have been underestimated. Nevertheless, we could show that abdominal obese men had greater muscle strength decline. Secondly, there were no information about participants’ dietary habits or their history of abdominal obesity. Such information could contribute to a better understanding of the effect of abdominal adiposity on the decline in muscle strength. Lastly, waist circumference does not provide direct estimates of visceral adiposity (pathogenic) such as computerized tomography or magnetic resonance imaging. However, it is a useful clinical screening tool.

In summary, abdominal obesity affects the muscle strength decline differently in men and women. Abdominal obese men are at greater risk of accelerated muscle strength decline. In addition, abdominal obesity defined by waist circumference seems to be a better measure to identify older adults at risk of muscle strength decline compared with BMI, especially in men. Our main findings highlight the importance of abdominal obesity in the prevention of muscle strength loss in older adults, especially in men. Because abdominal obesity can be controlled, its metabolic impact and associated costs to the loss of muscle strength can be prevented or reduced.

## Funding

This work was supported by the Brazilian Fostering Agency Coordination for the Improvement of Higher Education Personnel/Academic Excellence Programme (CAPES/PROEX). English Longitudinal Study of Ageing is funded by National Institute on Aging/National Institutes of Health (NIA/NIH) USA (grant R01AG017644) and by a consortium of the UK government departments coordinated by the Economic and Social Research Council (ESRC). The funders had no role in study design, data collection and analysis, or preparation of the manuscript.

## Supplementary Material

gly178_suppl_Supplemental_Table_1

gly178_suppl_Supplemental_Table_2

gly178_suppl_Supplemental_Table_3
