# Supplementary material for: Does Abdominal Obesity Accelerate Muscle Strength Decline in Older Adults? Evidence From the English Longitudinal Study of Ageing
Source: J Gerontol A Biol Sci Med Sci. 2018 Aug 10;74(7):1105–11. doi: 10.1093/gerona/gly178 (PMC6580692; doi:10.1093/gerona/gly178)
Supplement: gly178_suppl_Supplemental_Table_1 [file gly178_suppl_supplemental_table_1.docx]

Supplemental Table 1. Comparisons between included and excluded participants in ELSA at baseline

|  | Included (n=5,181) | Excluded (n=2,485) |
| --- | --- | --- |
| Sex (women), (%) | 54.6 | 55.7 |
| Age, years | 65.8 (9.4)* | 68.2 (10.2)* |
| Marital status (with conjugal life), (%) | 68.8* | 60.4* |
| Wealth (quintiles), (%) |  |  |
| Lowest quintile | 14.4* | 23.3* |
| 2^nd^ quintile | 18.3* | 21.6* |
| 3^rd^ quintile | 20.6* | 18.7* |
| 4^th^ quintile | 22.1* | 17.5* |
| Highest quintile | 23.3* | 17.5* |
| Not declared | 1.3* | 1.4* |
| Schooling, (%) |  |  |
| 0 to 11 years | 48.8* | 58.8* |
| 12 to 13 years | 25.2* | 20.4* |
| >13 years | 26.0* | 20.8* |
| Physical activity level, (%) |  |  |
| Sedentary | 3.4* | 11.0* |
| Low | 14.3* | 21.5* |
| Moderate | 51.6* | 46.1* |
| Vigorous | 30.7* | 21.4* |
| Alcohol intake, (%) |  |  |
| ≤1 day | 33.5* | 34.1* |
| 2 to 6 days | 41.4* | 31.2* |
| Daily | 16.7* | 14.0* |
| Not declared | 8.4* | 20.7* |
| Smoking, (%) |  |  |
| Non-smoker | 37.5* | 34.5* |
| Former smoker | 48.9* | 48.1* |
| Current smoker | 13.6* | 17.4* |
| Stroke (yes), (%) | 1.1* | 3.0* |
| Anaemia (yes), (%) | 5.3* | 8.6* |
| Cancer (yes), (%) | 2.7* | 4.0* |
| Heart disease (yes), (%) | 7.5* | 12.6* |
| Diabetes (yes), (%) | 4.2* | 6.0* |
| Arthritis (yes), (%) | 33.9* | 39.6* |
| Hypertension (yes), (%) | 43.2* | 36.7* |
| Osteoporosis (yes), (%) | 5.9 | 6.9 |
| Lung disease (yes), (%) | 13.6 | 14.3 |
| Fall (yes), (%) | 20.3* | 27.1* |
| Cognition (mean), points | 10.2 (3.4)* | 9.3 (3.8)* |
| Depressive symptoms (yes), (%) | 13.3* | 19.3* |
| Handgrip (kg),(mean) | 31.4 (11.5)* | 29.7 (12.0)* |
| Waist circumference (cm), (mean) | 95.0 (12.7)* | 97.2 (14.0)* |
| Abdominal obese (%) | 48.9* | 57.8* |
| Body mass index (kg/m²), (mean) | 27.7 (4.6)* | 28.4 (5.1)* |
| Normal weight (%) | 28.1* | 24.6* |
| Underweight (%) | 0.8* | 0.9* |
| Overweight (%) | 44.1* | 41.4* |
| Obese (%) | 27.0* | 33.1* |
| Triglycerides (≥150 mg/dl), (%) | 41.5* | 57.5* |
| Total cholesterol (≥200 mg/dl), (%) | 73.2 | 72.2 |
| HDL *(<*40 mg/dl ♂; <50 mg/dl ♀), (%) | 13.7* | 22.8* |
| LDL (≥100 mg/dl), (%) | 85.0* | 80.6* |
| C-reactive protein (>3 mg/l), (%) | 34.9* | 42.9* |
| Fibrinogen (>380 mg/dl), (%) | 16.7 | 18.7 |
| Ferritin (<39 ng/ml ♂; <62 ng/ml ♀), (%) | 20.6 | 18.8 |
| ADL (mean of impairments) | 0.3 (0.8)* | 0.6 (1.2)* |
| IADL (mean of impairments) | 0.3 (0.8)* | 0.7 (1.4)* |
| Data are presented as proportions, means and standard deviation. *Statistical significance was set as p<0.05. | | |
