# Supplementary material for: Does Abdominal Obesity Accelerate Muscle Strength Decline in Older Adults? Evidence From the English Longitudinal Study of Ageing
Source: J Gerontol A Biol Sci Med Sci. 2018 Aug 10;74(7):1105–11. doi: 10.1093/gerona/gly178 (PMC6580692; doi:10.1093/gerona/gly178)
Supplement: gly178_suppl_Supplemental_Table_2 [file gly178_suppl_supplemental_table_2.docx]

Supplemental Table 2. Estimated annual change on handgrip strength values over an 8-year follow-up period – (2004/05 – 2012/13)

|  | Women | | | | Men | | | |
| --- | --- | --- | --- | --- | --- | --- | --- | --- |
| Year | Non-Abdominal Obese | | Abdominal  Obese | | Non-Abdominal Obese | | Abdominal  Obese | |
|  | Predictive value (kg) | Decrease value (kg) | Predictive value (kg) | Decrease value (kg) | Predictive value (kg) | Decrease value (kg) | Predictive value (kg) | Decrease value (kg) |
| Intercept (baseline) | 24.23* | - | 25.07* | - | 40.34* | - | 41.91* | - |
| 1 | 23.94 | -0.29 | 24.76 | -0.31 | 39.89 | -0.45* | 41.34 | -0.57* |
| 2 | 23.66 | -0.28 | 24.44 | -0.32 | 39.44 | -0.45* | 40.77 | -0.57* |
| 3 | 23.37 | -0.29 | 24.13 | -0.31 | 38.99 | -0.45* | 40.19 | -0.58* |
| 4 | 23.08 | -0.29 | 23.82 | -0.31 | 38.54 | -0.45* | 39.62 | -0.57* |
| 5 | 22.80 | -0.28 | 23.50 | -0.32 | 38.09 | -0.45* | 39.05 | -0.57* |
| 6 | 22.51 | -0.29 | 23.19 | -0.31 | 37.65 | -0.44* | 38.47 | -0.58* |
| 7 | 22.22 | -0.29 | 22.88 | -0.31 | 37.20 | -0.45* | 37.90 | -0.57* |
| 8 | 21.93 | -0.29 | 22.56 | -0.32 | 36.75 | -0.45* | 37.33 | -0.57* |

*** Difference by abdominal obesity status within the same gender (p<0.05).
