# Supplementary material for: Does Abdominal Obesity Accelerate Muscle Strength Decline in Older Adults? Evidence From the English Longitudinal Study of Ageing
Source: J Gerontol A Biol Sci Med Sci. 2018 Aug 10;74(7):1105–11. doi: 10.1093/gerona/gly178 (PMC6580692; doi:10.1093/gerona/gly178)
Supplement: gly178_suppl_Supplemental_Table_3 [file gly178_suppl_supplemental_table_3.docx]

Supplemental Table 3. Generalized linear mixed models estimates for handgrip normalized to body mass index values as a function of abdominal obesity status over a 8-year follow-up period

|  | Women  n = 2,827 | Men  n = 2,354 |
| --- | --- | --- |
|  | Estimated Parameter (95%CI) |  |
| Intercept (baseline) |  |  |
| Non-abdominal obese | Reference | Reference |
| Abdominal obese | -0.118 (-0.134 – -0.103)** | -0.135 (-0.158 – -0.112)** |
| Slope (follow-up) |  |  |
| Time, years | -0.002 (-0.024 – 0.019) | -0.018 (-0.046 – 0.009) |
| Time x Non-abdominal obese | Reference | Reference |
| Time x Abdominal obese | 0.004 (0.001 – 0.008)* | 0.001 (-0.002 – 0.006) |

Note: CI: confidence interval; all models adjusted by socioeconomic variables, behavioural characteristics, health conditions, depression, cognition, serum markers and disability. *p<0.05; **p<0.01.
